# Wind Speed Perception and Risk

**DOI:** 10.1371/journal.pone.0049944

**Published:** 2012-11-30

**Authors:** Duzgun Agdas, Gregory D. Webster, Forrest J. Masters

**Affiliations:** 1 Engineering School of Sustainable Infrastructure & Environment, College of Engineering, University of Florida, Gainesville, Florida, United States of America; 2 Department of Psychology, College of Liberal Arts and Sciences, University of Florida, Gainesville, Florida, United States of America; CSIC-Univ Miguel Hernandez, Spain

## Abstract

**Background:**

How accurately do people perceive extreme wind speeds and how does that perception affect the perceived risk? Prior research on human–wind interaction has focused on comfort levels in urban settings or knock-down thresholds. No systematic experimental research has attempted to assess people's ability to estimate extreme wind speeds and perceptions of their associated risks.

**Method:**

We exposed 76 people to 10, 20, 30, 40, 50, and 60 mph (4.5, 8.9, 13.4, 17.9, 22.3, and 26.8 m/s) winds in randomized orders and asked them to estimate wind speed and the corresponding risk they felt.

**Results:**

Multilevel modeling showed that people were accurate at lower wind speeds but overestimated wind speeds at higher levels. Wind speed perceptions mediated the direct relationship between actual wind speeds and perceptions of risk (i.e., the greater the perceived wind speed, the greater the perceived risk). The number of tropical cyclones people had experienced moderated the strength of the actual–perceived wind speed relationship; consequently, mediation was stronger for people who had experienced fewer storms.

**Conclusion:**

These findings provide a clearer understanding of wind and risk perception, which can aid development of public policy solutions toward communicating the severity and risks associated with natural disasters.

## Introduction

Wind is the primary agent for two of the most destructive natural hazards on earth—hurricanes and tornadoes. Storm preparation, evacuation, and hazard mitigation depend significantly on risk perception [1], [2], and effective policy making and implementation necessitates understanding human perception of hazards and associated risks [3]. Prior experimental research on wind–human interaction has focused on pedestrian ‘comfort’ in urban areas [4]–[6] by establishing wind speed thresholds that make daily tasks challenging, uncomfortable, or cause people to feel unsafe [7]. Findings were largely based on two-choice semantic responses (e.g., gentle-violent, calm-gale, pleasant-annoying [4], [8]) or characterizations of physical responses (e.g., loss of balance, shifts in footstep trajectories [9], [10]). Surprisingly, however, empirical research is lacking on (a) people's accuracy in perceiving wind speed while they are experiencing it and (b) people's perception of personal risk in response to wind. Understanding people's perceptual accuracy of extreme wind speeds is important because storms often cause massive power and communication disruptions that leave people without official weather warnings or reports. The effectiveness of weather warnings in conveying the actual risks associated with extreme wind events may be suboptimal even if information regarding wind speed severity is made available [3], [11]. Our goal in the present experiment was to address these shortcomings by exposing people to various wind speeds to gain a better understanding of wind and risk perception—factors that could be key in developing better policy and warning systems for extreme wind-related events. This might include supplementing extreme wind-related warnings by framing them in familiar contexts (e.g., “a wind of this speed or greater is enough to knock over a person”).

This present experiment examined human perception of extreme winds and associated risks. Because prior research in established perceptual domains (e.g., vision, audition, just noticeable differences in weight perception) has shown human perception to be a nonlinear transform of physical stimuli (e.g., Weber–Fechner law), we predicted that people would overestimate wind speeds at higher velocities, and that risk perceptions would follow a similar accelerating trajectory. We also expected that wind speed perception would mediate the relationship between actual wind velocity and perceptions of risk; overestimates of wind speed would relate to more perceived risk. On an exploratory basis, we also examined the extent to which individual differences in prior storm experience moderated these relationships.

## Methods

### Ethics Statement

Ethical standards outlined by the American Psychological Association were followed in the conduct of this research, which was approved by the University of Florida Institutional Review Board. All participants gave their signed consent prior to partaking in the experiment.

### Participants and Procedure

Seventy-six college-age students (18 women, 58 men) aged 18 to 40 years (*M* = 23.47, *SD* = 4.68) participated in the study. Participants were first given surveys on their prior experiences with and beliefs about extreme weather phenomena and associated decision-making. Next, participants donned protective gear (goggles, waders, and hooded raincoats) and a harness that attached to a handrail system located 8 ft downwind of the jet, which they were allowed to hold. Participants were then exposed to 10, 20, 30, 40, 50, and 60 mph (4.5, 8.9, 13.4, 17.9, 22.3, and 26.8 m/s) wind speeds for 20-s intervals in predetermined randomized orders (see Video S1). In between each wind exposure event (which lasted ≈10 s), participants communicated their estimate of the wind speed and their estimate of personal injury risk on a scale of 0 (*no perceived risk*) to 10 (*dangerous*) to an observer standing outside the wind field. Thus, the total experiment duration was ≈3 min for each participant. The testing conditions (wind speed intensities, total exposure time, gear) were identical for all participants; only the order of wind speeds was randomized to control for possible order effects. Participants were given no information on the wind speed intensities to prevent possible bias; however, they were informed of the wind speeds after the experiment.

### Wind Apparatus

**Figure 1:**
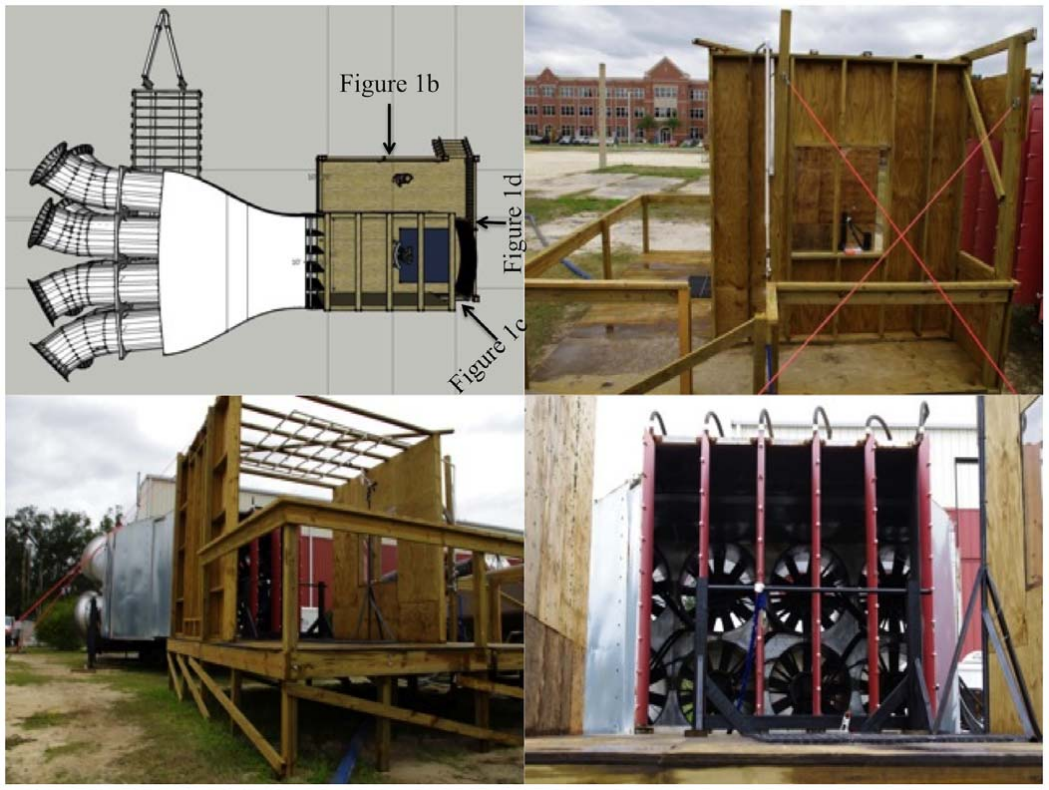
Design and photographs of the wind simulator. The upper left panel shows the wind simulator's design; the other three panels show photographs of it from different angles.

Eight 54-in (1.37-m) diameter vaneaxial fans forced air through a 10-ft×10-ft (3.05-m×3.05-m) square jet to generate the wind field in the test chamber (Figure 1). Hydraulic power to the fans was individually controlled to regulate the angular velocity of the fans to reach a desired flow. An RM Young Wind Monitor located in the test chamber measured wind speed, which was read by the equipment operator.

### Data Analysis

Because repeated estimates were nested within participants, we analyzed the data with multilevel modeling (MLM [12], [13]) using HLM [14] and Mplus [15]. Using maximum likelihood estimation, MLM can model within- and between-person effects simultaneously. Within-person (or between-trial) variance in wind or risk perception was modeled at level 1, and between-person variance was modeled at level 2 as a function of between-person means (intercepts) and, in some models, individual differences in number of tropical cyclones experienced (i.e., the tropical storms with sustained winds ≥39 mph or 63 km/h, hereafter referred to in shorthand as “storms”). For example, in one analysis we modeled wind speed perception as a function of actual wind speed (level 1) and number of storms experienced (level 2). The level-1 model was:
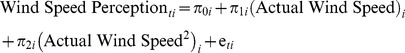
(1)where Perception*_ti_* represents the wind speed estimate for Speed *t* by Person *i*. Each person's Perception scores are modeled as functions of their mean or intercept (π_0*i*_) and the linear (π_1*i*_) and quadratic (π_2*i*_) effects of actual wind speed. The error term, *e_ti_*, captures the level-1 residual variance for each person.

In MLM, the level-1 intercepts and slopes for each person are modeled at level 2 as a function of individual differences in the number of tropical cyclones experienced (grand-mean-centered at 5.05 storms):
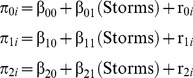
(2)Here, π_0*i*_ again represents the mean (intercept) for each person. The β_00_ coefficient represents the grand mean—the between-person average of each person's average intensity score—for the average number of storms experienced. The coefficients β_10_ and β_20_ represent the between-person average of the within-person linear and quadratic effects (respectively) of actual wind speed on wind speed perceptions. The coefficients β_01_, β_11_, and β_21_ represent the extent to which the within-person intercepts and linear and quadratic effects (respectively) are moderated by individual differences in the number of tropical cyclones people have experienced. The error terms *r*_0*i*_, *r*_1*i*_, and *r*_2*i*_ capture the level-2 residual variance for their respective effects.

In the multilevel moderated mediation models below, this MLM framework is expanded to include mediation at level 1 with a continuous level-2 moderator (number of tropical cyclones experienced). We followed procedures outlined in prior work [16]–[18].

## Results

### Descriptive Statistics

Table 1 lists descriptive statistics for perceived wind speed and risk by actual intensity.

**Table 1:** Descriptive statistics for wind and risk perceptions by actual wind speed.

	Perceived Wind Speed (mph)						Perceived Risk on a 1 to 10 Scale						
Actual Wind Speed mph (m/s)	Range	*Mdn*	Mean	*SD*	Skew.	Exc. Kurt.	Range	*Mdn*	Mean	*SD*	Skew.	Exc. Kurt.	*r*
10 (4.5)	1–60	10.0	10.2	8.2	3.42	18.05	0–5	1.0	0.81	0.87	1.84	6.12	.15
20 (8.9)	4–40	20.0	20.6	9.3	0.51	−0.28	0–5	2.0	1.68	1.12	0.43	−0.17	.45*
30 (13.4)	10–75	30.0	33.7	13.6	0.58	0.21	0–7	3.0	3.21	1.54	−0.03	−0.23	.48*
40 (17.9)	10–90	45.0	45.2	17.5	0.43	−0.31	1–9	4.0	4.46	1.69	0.66	1.23	.72*
50 (22.3)	15–115	57.5	60.4	19.4	0.55	0.30	3–10	6.0	5.99	1.79	0.26	−0.51	.45*
60 (26.8)	30–130	75.0	75.8	25.4	0.22	−0.50	2–10	8.0	7.34	1.89	−0.57	−0.18	.65*

*Note.* Nesting *not* taken into account for this table; data averaged across persons rather than examining data within persons. Skew. = Skewness. Exc. Kurt. = Excess Kurtosis. *r* = correlation between wind perceptions and risk perceptions. *N*s = 76 participants, 454 observations (2 data points missing due to procedural error).

* *p*<.05.

### Wind Speed Perception as a Function of Actual Wind Speed

**Figure 2:**
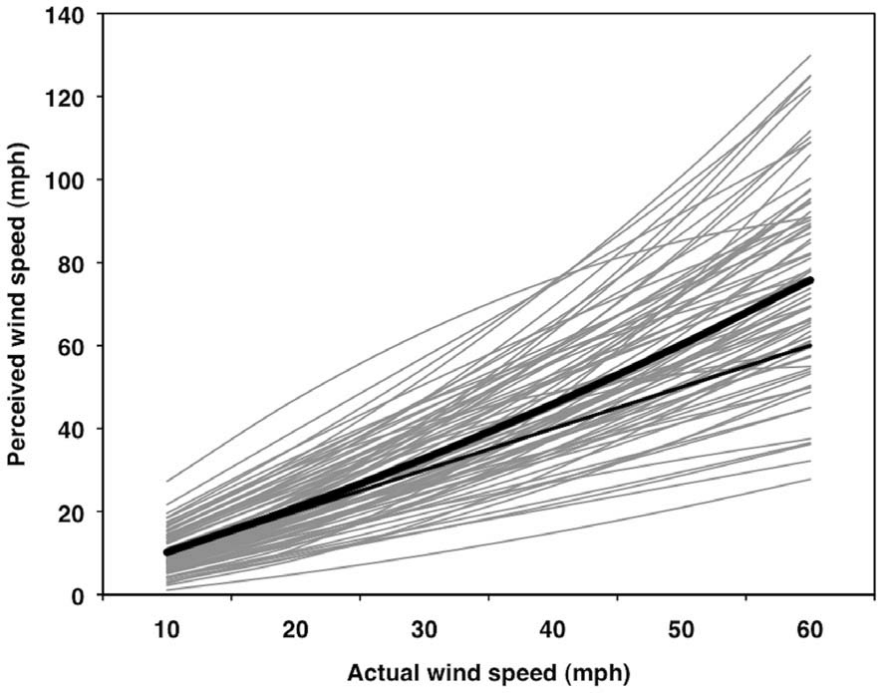
Multilevel modeling results for perceived wind speed as a function of actual wind speed. Thin gray lines represent individual predicted curves for 76 participants. Thick black line represents the average curve. Thin black line represents a one-to-one relationship.

**Figure 3:**
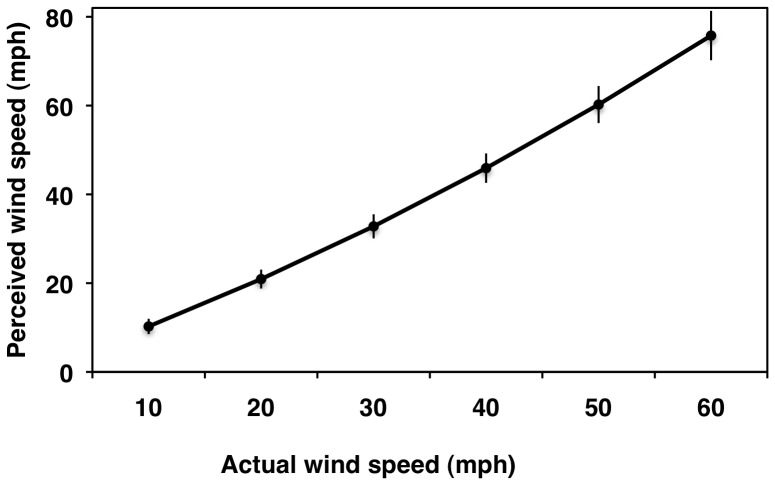
Perceived wind speed as a function of actual wind speed. Point estimates and 95% confidence intervals are shown for the average slope for each wind speed tested.

Both the linear (β_10_ = 1.311, *SE* = 0.054, *t*_75_ = 24.23, partial correlation [*r*_p_] = .94) and quadratic (β_20_ = 0.0061, *SE* = 0.0016, *t*_75_ = 3.80, *r*_p_ = .40) effects of actual wind speeds on people's perceptions of wind speeds were significantly positive for the average person (*p*s<.05; Figures 2 and 3). For this model, 76% and 24% of the variance was at the between- and within-person levels, respectively. The average person was reasonably accurate and perception was fairly linear at slower wind speeds, but the perceived wind speeds departed from both accuracy and linearity at higher wind speeds.

**Figure 4:**
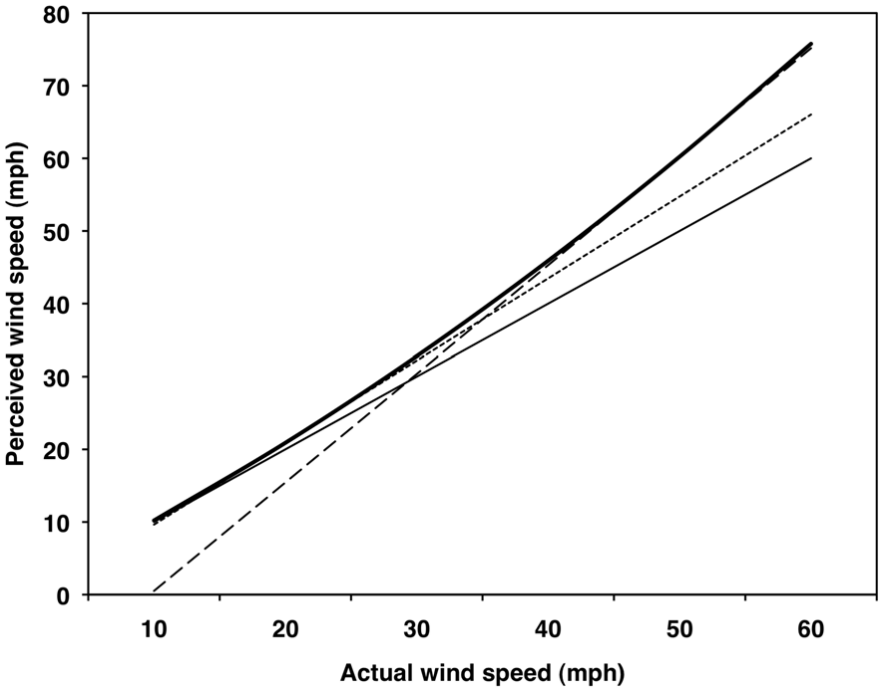
Perceived wind speed as a function of actual wind speed: Simple slopes. Examples of simple slopes tangent to the average curve (thick solid line) at 20 (dotted line) and 50 (dashed line) mph (8.9 and 22.3 m/s). Slopes are shown in reference to a one-to-one relationship (thin solid line).

Simple effects tests [19] showed that the average perception did not differ significantly from actual wind speeds at 10 and 20 mph (4.5 and 8.9 m/s); however, beginning at 30 mph (13.4 m/s), the average person progressively overestimated the actual wind speeds (Table 2, left; Figures 2 and 3). We also tested the extent to which the average perceptions fit or departed from a one-to-one accuracy slope across the six wind speeds. The simple slope between perceived and actual wind speeds was computed for each one of the six wind speed levels (i.e., 10–60 mph; 4.5–26.8 m/s). This is equivalent to asking whether the lines tangent to the curve at each one of the six speeds is significantly different than the one-to-one line (Figure 4). At 10 mph (4.5 m/s), the simple slope was not significantly different from a one-to-one relationship; however, starting at 20 mph (8.9 m/s), the simple slopes were significantly more positive than the one-to-one relationship, suggesting that people became less accurate about the wind function (departed from linearity) as wind speeds increased (Table 2, right), which is to be expected as the wind forces exerted on the human body are proportional to the wind speed squared.

**Table 2:** Simple effects: Wind perception as a function of actual wind speed.

Actual Wind Speed mph (m/s)	Intercept (difference from actual)				Slope (difference from one-to-one)			
	β_00_	*SE*	*t*_75_	*d*	β_10_	*SE*	*t*_75_	*d*
10 (4.5)	0.251	0.880	0.28	0.08	0.006	0.071	0.08	0.02
20 (8.9)	0.920	1.090	0.84	0.22	0.128	0.052	2.48*	0.66
30 (13.4)	2.798	1.385	2.02*	0.54	0.251	0.048	5.19*	1.37
40 (17.9)	5.910	1.693	3.49*	0.92	0.372	0.0635	5.85*	1.55
50 (22.3)	10.234	2.130	4.80*	1.27	0.493	0.088	5.59*	1.48
60 (26.8)	15.770	2.839	5.55*	1.47	0.614	0.116	5.28*	1.40

*Note. N*s = 76 participants, 454 observations (2 data points missing due to procedural error).

* *p*<.05.

### Risk as a Function of Actual Wind Speed

**Figure 5:**
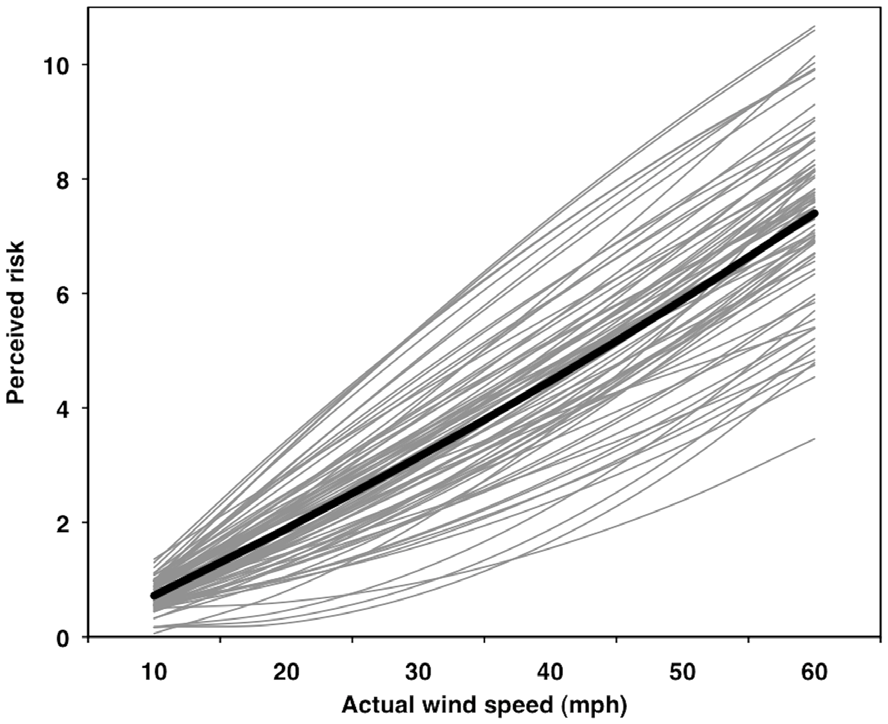
Multilevel modeling results for perceived risk as a function of actual wind speed. Thin gray lines represent individual predicted curves for 76 participants. Thick black line represents the average curve.

Both the linear (β_10_ = 0.1336, *SE* = 0.0045, *t*_75_ = 30.01, *r*_p_ = .96) and quadratic (β_20_ = 0.00043, *SE* = 0.00020, *t*_75_ = 2.14, *r*_p_ = .24) effects of wind speed on people's perceptions of risk were both significantly positive for the average person (*p*s<.05; Figure 5). A slope of 0.1336 in Model 1 indicates that, for every 10-mph (4.5-m/s) increase in wind speed, the average participant's perception of risk increase 1.336 units on a 0–10 scale. For this model, 67% and 33% of the variance was at the between- and within-person levels, respectively. The average person's risk function for actual wind speeds was curvilinear and concave up (accelerating; Figure 5). This trend was supported via a series of simple effects tests at each wind speed; for example, the simple slopes at 10 and 60 mph (4.5 and 26.8 m/s) were 0.112 (*SE* = 0.011, *t*_75_ = 9.80, *r*_p_ = .75) and 0.155 (*SE* = 0.011, *t*_75_ = 14.66, *r*_p_ = .86), respectively (*p*s<.05).

### Risk as a Function of Wind Perception

**Figure 6:**
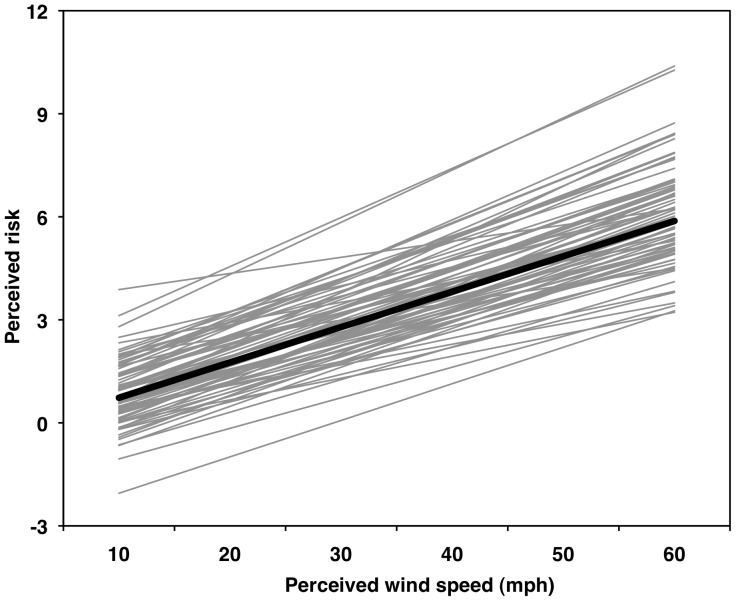
Multilevel modeling results for perceived risk as a function of perceived wind speed. Thin gray lines are individual predicted curves for 76 participants. Thick black line represents the average curve.

Perceptions of wind and risk were linearly related (β_10_ = 0.1031, *SE* = 0.0035, *t*_75_ = 29.46, *p*<.05, *r*_p_ = .96); no significant quadratic effect was present (*r*_p_ = −.06; Figure 6). A slope of 0.1031 indicates that, for every 10-mph (4.5-m/s) increase in perceived wind speed, the average participant's perception of risk increased about 1.031 units on a 0–10 scale. For this model, 65% and 35% of the variance was at the between- and the within-person levels, respectively.

### Number of Storms Experienced Moderates the Actual–Perceived Wind Relationship

**Figure 7:**
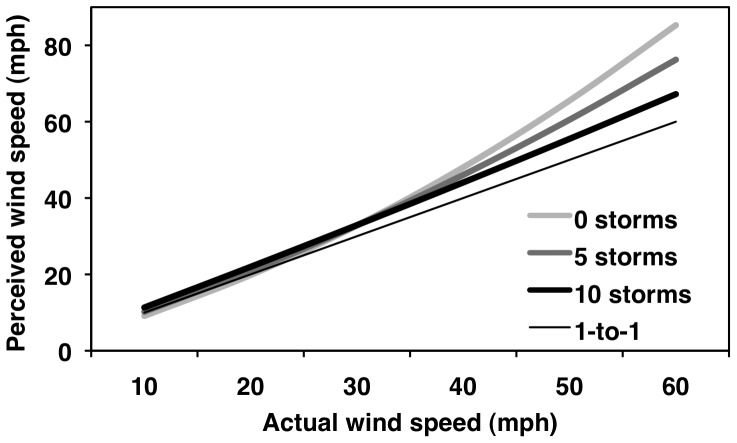
Perceived wind speed as a function of actual wind speed and number of storms experienced.

We tested the extent to which individual differences in the number of tropical cyclones people had experienced (*n* = 75; range: 0–10-or-more; *Mdn* = 5.0, *M* = 5.0, *SD* = 3.0) moderated the within-person actual–perceived wind speed relationships. The purpose was to determine whether the number of tropical storms people had experienced relates to the average actual–perceived wind speed relationship. Number of storms experienced significantly moderated the linear (β_11_ = −0.037, *SE* = 0.015, *t*_73_ = −2.48, *p*<.05, *r*_p_ = −.28) but not the quadratic (β_21_ = −0.00078, *SE* = 0.00049, *t*_73_ = −1.57, *p* = .12, *r*_p_ = −.18) effect of actual wind speed on wind perceptions (Figure 7). (It is unlikely that four participants experienced 10 or more tropical cyclones based on their age and historic data. The reported exposure inaccuracies might relate to misperceptions about the environmental conditions that constitute tropical cyclones. Nevertheless, when we re-ran the model without these four participants, number of storms experienced still moderated the linear effect of actual wind speeds on wind speed perception, β_11_ = −0.034, *SE* = 0.016, *t*_69_ = −2.01, *p*<.05, *r*_p_ = −.23).

We decomposed this model by conducting simple effect tests at the minimum (0) and maximum (10) reported values for number of tropical cyclones experienced. For people who experienced no storms, both the linear (β_10_ = 1.50, *SE* = 0.10, *t*_73_ = 14.70, *r*_p_ = .86) and quadratic (β_20_ = 0.0101, *SE* = 0.0029, *t*_73_ = 3.52, *r*_p_ = .38) effects of actual wind speeds on perceived wind speed were significant (*p*s<.05); moreover, people's average linear slopes were significantly different from a one-to-one relationship (β_10_ = 0.50, *SE* = 0.10, *t*_73_ = 4.93, *p*<.05, *d* = 1.15; Figure 7, thick light-gray curve). In contrast, for people who experienced ten or more storms, the relationship was strictly linear (β_10_ = 1.130, *SE* = 0.081, *t*_73_ = 14.00, *p*<.05, *r*_p_ = .85)—the quadratic effect (β_20_ = 0.0023, *SE* = 0.0030, *t*_73_ = 0.78, *p* = .44, *r*_p_ = .09) was non-significant; moreover, people's average linear slopes did not differ significantly from a one-to-one relationship (β_10_ = 0.130, *SE* = 0.081, *t*_73_ = 1.61, *p* = .11, *d* = 0.38; Figure 7, thick black curve). On an exploratory basis, we also tested the simple moderation effect of number of storms experienced on wind perception at 60 mph (26.8 m/s). At 60 mph (26.8 m/s), number of storms experienced marginally (i.e., *p*<.06) moderated people's perceptions of wind speed (β_01_ = −1.65, *SE* = 0.85, *t*_73_ = −1.95, *p* = .055, *r*_p_ = −.22; Figure 7, rightmost ends of curves).

### Wind Perception Mediates the Wind–Risk Relationship and Strengthens with Inexperience

**Figure 8:**
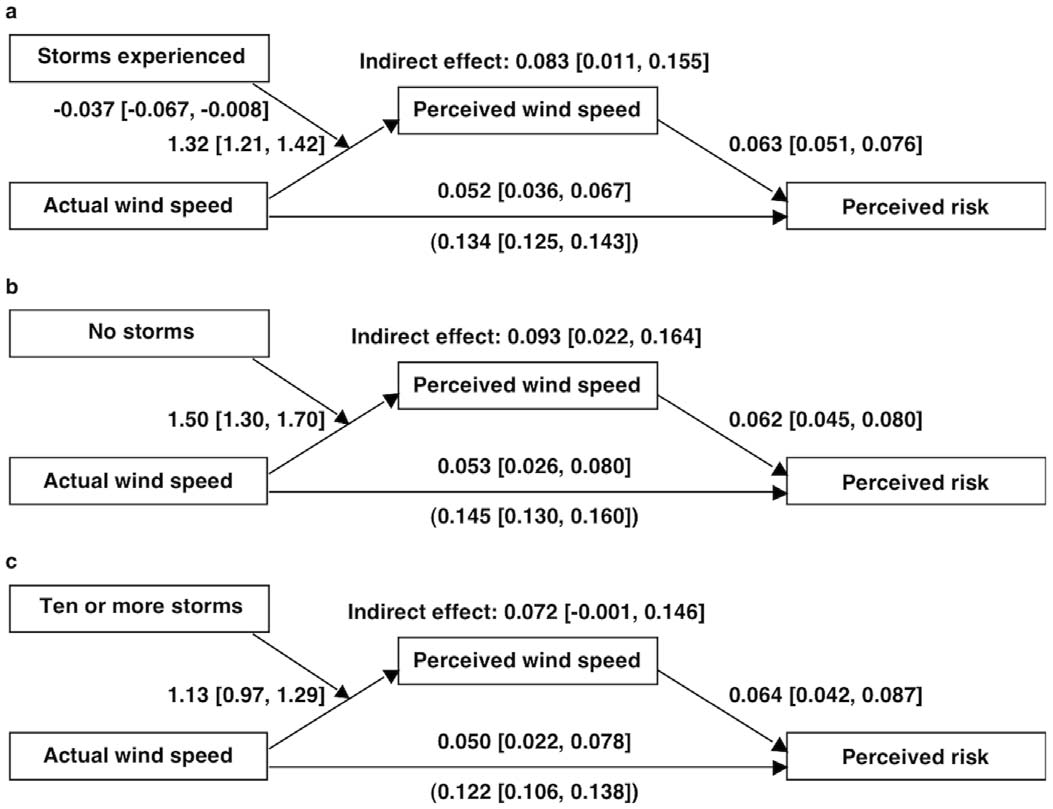
Multilevel moderated mediation model results. *Panel A* shows a moderated mediation model showing that (a) perceived wind speed (mph) partially but significantly mediated the relationships between actual wind speed (mph) and perceived risk (linear relationships only) and (b) number of storms experienced (grand-mean centered) moderated the relationship between actual and perceived wind speed. *Panels B* and *C* show simple effects tests of the mediation model at zero and ten-or-more storms experiences, respectively. Values are unstandardized regression coefficients [95% CIs]. The direct relationship between actual wind speed and perceived risk is shown in parentheses.

We tested a multilevel moderated mediation model (mediation at the lower level, moderation at the upper level [16], [17]) to assess (a) if wind perceptions mediated the direct relationship between actual wind and risk perceptions and (b) if individual differences in experience with tropical cyclones moderated the strength of the mediation. We tested only linear effects because (a) they were substantially stronger than the quadratic effects and (b) the quadratic effect of actual wind speed on risk was non-significant after controlling for wind perceptions. Because all direct effects remained significant, all results showed partial (vs. complete) mediation. As shown in Figure 8a, when assessed at the mean number of tropical cyclones experienced, the direct relationship between actual wind and risk was significantly attenuated after controlling for wind perception; the indirect effect via wind perception was significant. The direct and indirect effects accounted for 38% and 62% of the total effect, respectively.

We next tested the strength of the mediation (via simple effects tests) for people who had experienced no storms or 10-or-more storms (Figures 8b and 8c, respectively). People who had experienced no storms had an especially strong actual–perceived wind relationship and showed a significant mediation pattern. The direct and indirect effects accounted for 36% and 64% of the total effect, respectively. In contrast, people who had experienced 10 or more storms had a weaker—but more accurate (their average slope did not differ significantly from a one-to-one relationship)—actual–perceived wind relationship and showed a non-significant mediation pattern, because the 95% CI for the indirect effect included zero (Figure 8c). The direct and indirect effects accounted for 41% and 59% of the total effect, respectively.

## Discussion

We began by asking how accurately people perceive extreme wind speeds and how their estimates affect their perceptions of personal risk. These are key questions for both the psychology of human perception and public policy in response to extreme wind-related weather events. The study results indicate that on average (a) people overestimate higher wind speeds (≥20 mph or 8.9 m/s) but are reasonably accurate at judging lower wind speeds, (b) the simple relationship between wind speed and perceived risk becomes increasingly positive at higher wind speeds, (c) wind perception mediates the relationship between actual wind speed and risk, and (d) this mediation pattern is stronger among people with no prior experience with tropical cyclones and weakens with exposure to each additional storm.

The new knowledge generated by this research is useful not only because it expands our understanding of how people perceive wind and wind-related risk on a psychological level, but also because it has potentially life-saving public policy implications on how information is communicated prior to and during extreme weather events (e.g., tornadoes, hurricanes). Although the average person overestimates higher wind speeds (e.g., they perceive 60-mph [26.8-m/s] winds to be 75 mph [33.5 m/s]), this relationship is moderated by individual differences in storm exposure; with each addition tropical cyclone people experienced, people made more accurate wind speed estimates on average. This suggests that exposure to real storms may help calibrate people's perceptions regarding higher wind speeds. With some exposure, people may be able to gauge wind speeds more accurately. Future research should strive to examine the processes by which individual differences in perceptual and risk judgments form. Nevertheless, our results also highlight a disconnect between wind perception and reality, perhaps because of people's lack of exposure to high-velocity wind speeds. For example, in Florida—the most hurricane-prone state in the U.S.—many coastal residents hail from outside the state [20] and thus have no prior experience with landfalling hurricanes. The findings indicated that people who have not experienced sustained tropical storm or hurricane-force winds are more prone to overestimating wind speed, which may negatively affect their decision-making about preparation and evacuation. For example, a major civil problem with government-issued evacuations is the phenomenon of “shadow evacuation,” in which people who do *not* need to evacuate chose to do so anyway, thereby unnecessarily exacerbating traffic jams along evacuation routes, and filling limited spaces in shelters and hotel rooms [21]. Further research is required to validate these findings, not only with a more diverse sample, but also with a more specific measure of risk that can distinguish between probability and severity.

Although additional research needs to be done before we can make any strong recommendations regarding public policy, the present research may suggest the possibility for introducing a risk metric or contextual aid to characterize wind speeds in storm advisories, particularly given that the difference between perceived and actual wind speed can be shown to be interpreted as a difference of one or two categories on the Saffir–Simpson Hurricane Wind Scale—a five-category classification system for hurricane intensity based on wind velocity. Such a dual system is used for hail advisories, where the U.S. National Weather Service reports both hail diameter information (in fractional inches) and the size of a common object (e.g., “dime-sized,” “quarter-sized,” “softball-sized”). Perhaps wind speeds could be accompanied by relevant information such as “this wind speed is sufficient to knock over the average person.” Nevertheless, we caution that the present research is preliminary, and additional research that focuses on public policy applications will need to be undertaken before any recommendations can be made. We hope our novel experimental findings on wind perception will inform not only the psychology of risk but also future research on the broader policy implications extreme weather preparation and response.

## Supporting Information

Video S1 **This video shows participants being exposed to various wind speed in the wind simulator (see Method section of text for details).** In this article, participants were exposed to dry winds; however, for better visualization, this video shows wind-driven rain, which was applied separately for a companion study.
(MOV)
